# Conductive Education for Children With Cerebral Palsy: A Systematic Review of Outcomes, Practice Time and Motor Performance Assessment

**DOI:** 10.1111/cch.70149

**Published:** 2025-07-31

**Authors:** Nathália Nídia da Silva, Wivianne Abreu Cavalcante, Albert Lucas Olinto Tertuliano, Alana Amicilene Azevedo de Sousa, Debora Chayeny Alves de Oliveira, Ariane Brito Diniz Santos, Anderson Henry Pereira Feitoza, Lorena Moraes Dantas, Marisete Peralta Safons, Maria Teresa Cattuzzo

**Affiliations:** ^1^ Higher School of Physical Education University of Pernambuco Recife Pernambuco Brazil; ^2^ Recife City Hall Recife Pernambuco Brazil; ^3^ University of Brasilia Brasilia Distrito Federal Brazil

**Keywords:** child development, motor disorders, physical functional performance

## Abstract

**Aim:**

To investigate Conductive Education (CE) interventions in children with cerebral palsy (CP), examining how practice time, assessment methods, and CP characteristics influence treatment outcomes.

**Method:**

A systematic review (PROSPERO‐CRD42024578760) searched seven databases using ‘Conductive Education’. Inclusion criteria: interventional studies in young people with CP receiving CE treatment. The PRISMA strategy guided study selection, aided by Rayyan software. Study quality was assessed using ROBINS‐I.

**Results:**

Eighteen studies were included. Seven studies showed low risk of bias; considering low and moderate risk studies, 67% demonstrated positive CE effects. Practice duration appeared crucial: Studies reporting positive outcomes averaged 25.2 h/week compared to 17.7 h/week in studies showing no effect. The Gross Motor Function Measure was the most used assessment tool, followed by the Paediatric Evaluation of Disability Inventory. CE showed better outcomes in spastic CP, particularly in cases with diplegic presentation, compared to athetoid or ataxic types.

**Interpretation:**

CE demonstrates promise for improving motor performance in children with CP, particularly with adequate practice time (≈25 h/week). Treatment success appears influenced by CP type and assessment methods. Future research should prioritize standardized protocols and consistent outcome measures to strengthen evidence quality.

## Summary

- This study offers a critical examination of the methodological aspects of CE studies.
- This study provides a comprehensive synthesis of the CE research.
- CE is a promising intervention to increase motor performance in youth with CP.
- This study aims to contribute to the improvement of evaluation practices in this field.
- Practice time deserves to be better considered in future studies with CE.

## 1 Introduction

Conductive education (CE) emerged in 1948 as a novel approach to rehabilitate children with cerebral palsy (CP) who were often excluded from mainstream education due to their motor disabilities. Developed by András Petö in Hungary, CE is based on the premise that children with neuromotor disorders can learn to perform daily living skills through appropriate instruction, motivation and practice (Bourke‐Taylor et al. 2007; Sutton 1988).

CE aims to replace the concept of dysfunction with ‘orthofunction’, focusing on enhancing motor, social, intellectual and psychological performance in individuals with neuromotor disorders (Coles and Zsargo 2003). This method employs active learning and neuroplasticity principles to mitigate the physical effects of various neuromotor disabilities, including CP, spina bifida, multiple sclerosis, Parkinson's disease and acquired brain injury (Bourke‐Taylor et al. 2007).

The CE approach is characterized by group or individual training sessions facilitated by a specially trained conductor. It incorporates adapted equipment and rhythmic intention techniques to promote motor independence and self‐efficacy (Myrhaug et al. 2019). The programme integrates individual characteristics, task demands and environmental factors to improve motor control and enhance cognitive, motor and sensory‐perceptive system interactions (O'Shea et al. 2020).

From an initial literature review on this topic, it was observed that the quality of CE studies has been criticized due to methodological biases that may compromise research outcomes (Myrhaug et al. 2014; Novak et al. 2013). Researchers have highlighted difficulties in assessing motor progress in children with CP undergoing CE, citing inconsistencies between examiners and limitations in the validity and reliability of assessment tools (Sigafoos et al. 1994; Catanese et al. 1995). In children with CP, the type and topography deserve to be considered. Also, insufficient practice time is a potential source of poor interpretation in intervention studies. Therefore, the aim of this review is to describe the interventional studies of CE in children with CP, specifically observing the methodological quality of the studies, as well as the time of practice, the type of assessment and the type and topography of the palsy as possible variables to impact the studies.

## 2 Method

This is a systematic review that follows the guidelines of the Preferred Reporting Items for Systematic Reviews and Meta‐Analyses—PRISMA (Page et al. 2022). The review protocol was registered at PROSPERO (CRD42024578760).

### 2.1 Search Strategy

The search was conducted using studies published up to December 2023, with children aged 3 to 17 in the following databases: Pubmed, Science Direct, Embase, Web of Science, Scopus, Cochrane Library, Scielo and Sage. The PICO strategy was established to guide the searches: P = Children and adolescents with CP; I = Intervention using the CE method; C = Control group; O = Motor performance. After a series of initial searches, only the term ‘conductive education’ was adopted for the final search, since this is the specific term to describe the desired variable.

### 2.2 Inclusion/Exclusion Criteria

The following inclusion criteria were used: (a) intervention articles published in English, Portuguese and Spanish; (b) the population: children or adolescents with CP; (c) use of CE as a rehabilitation method. Those who were excluded (a) did not evaluate motor performance.

After the search, the articles were exported to the (free) web application Rayyan (https://rayyan.qcri.org), which assists in article screening. Initially, duplicate articles were removed. Next, the researchers (NNS, LMD) in double‐blind mode read the titles and abstracts to decide on the inclusion of articles in this review study. The included articles were reviewed by reading them in full (NNS, ALOT, AAAS, and DCAO) independently by the researchers. Finally, inconsistencies were evaluated and, if necessary, decided by a third researcher (MTC).

### 2.3 Data Extraction and Analysis

The data extracted from the studies will be related to the sample, evaluation used to measure the children's motor performance, intervention details and outcome. Data analysis begins with the article selection process. After that, absolute and relative frequency values for the variables were generated from the extracted data; the number and duration of sessions permitted calculating the week time of intervention. The critical analysis will be based on these synthesis data and the examination of the various tools used to evaluate motor performance in CE studies, as well as the type and topography of CP. The data compilation and statistical analysis were performed utilizing Microsoft Excel.

### 2.4 Studies Quality Assessment

The quality of the studies was assessed using the Risk Of Bias In Non‐randomized Studies of Interventions (ROBINS‐I) (Sterne et al. 2016). This tool was developed to assess the risk of bias in estimates of intervention effectiveness in studies that did not use randomization to allocate participants. The risk of bias is evaluated based on seven items, organized into three main domains: pre‐intervention, intervention and post‐intervention. Each domain is assessed individually, and the overall risk of bias for the study is determined based on the evaluation of these different aspects. The risk of bias is classified qualitatively in each domain with the following categories: low risk of bias (the study is judged to be at low risk of bias in all domains); moderate risk of bias (the study is judged to be at low or moderate risk of bias in all domains); serious risk of bias (the study is judged to be at serious risk of bias in at least one domain, but not at critical risk of bias in any domain); and no information (there is no clear indication that the study is at serious or critical risk of bias, but there is a lack of information in one or more key domains of bias). Only studies with low and moderate risk of bias will be considered in evaluating the intervention's effectiveness.

## 3 Results

**FIGURE 1:**
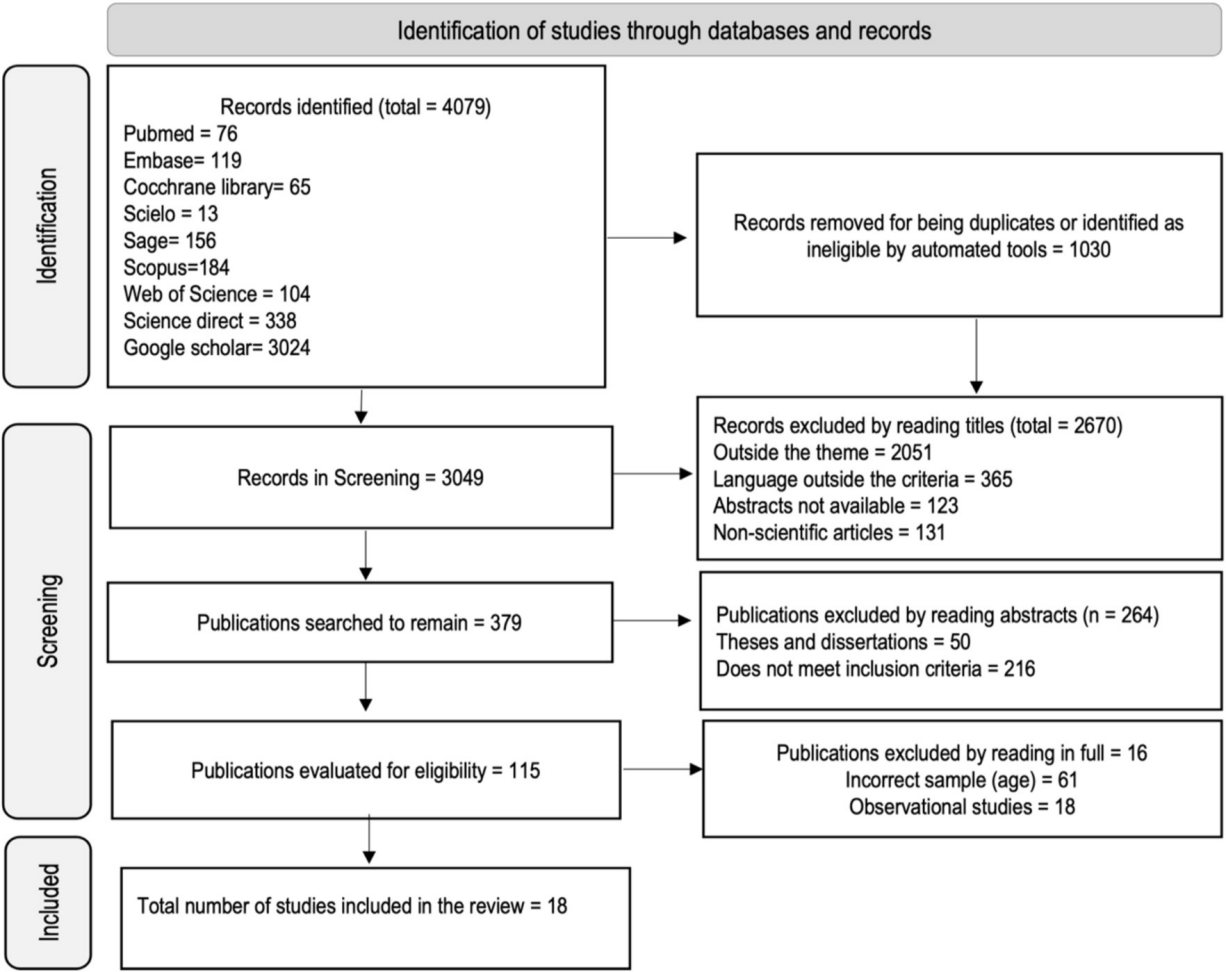
Flowchart of article inclusion using the PRISMA strategy. *Source:* Created by the author.

The article selection process from seven databases and Google Scholar resulted in eighteen included articles (Figure 1).

Most studies (90%) investigated children in early and middle childhood, with a mean age of 5.4 years (SD = 2.8). Only in the studies by Ödman and Öberg (2005, 2006) the ages of the participants ranged from 3 to 16 years. The data in Table 1 demonstrate how studies of CE are spread throughout the world. It is also evident that studies with the CP population are done with small or modest sample sizes.

**TABLE 1:** Descriptive analysis of the studies' characteristics.

	*N*	*f*
Year of publication of studies		
Years		
≤ 2000	4	22.2
2001–2010	9	50.0
2011–2020	5	28.0
Total	18	100
Country of origin		
Australia	4	22.2
Iran	2	11.1
China	2	11.1
Norway	2	11.1
Sweden	2	11.1
Germany	1	5.6
United Kingdom	1	5.6
Spain	1	5.6
New Zealand	1	5.6
USA	1	5.6
Canada	1	5.6
Total	18	100.0
Number of participants in studies' samples		
1	1	5.6
02 to 10	3	16.7
11 to 20	1	5.6
21 to 30	6	33.3
31 to 40	2	11.1
41 to 50	2	11.1
> 51	3	16.7
Total	18	100.0

Regarding the number and duration of sessions (Table 2), seven studies did not provide information about the duration and number of sessions. Observing the six studies with a positive effect of CE that reported the duration of practice, the average time was 25.2 h/week, ranging from a minimum of 9 to 40 h per week. On the other hand, observing the seven studies in which CE had no effect, but that reported the practice time, this time ranged from 2 to 24 h per week, with an average of 17.7 h/week of CE practice.

**TABLE 2:** Effect from EC and the quantity of practice.

	Daily session (hours)	Weekly sessions (days)	Hours/week
**Studies with positive effect from EC**			
Effgen and Chan (2010 )	8	5	40
Fase A (Blank et al. 2008)	7	5	35
Ilundáin and Fernández (2007 )	7	5	35
Coleman et al. (1995 )	4	5	20
Dalvand et al. (2009 )	3	4	12
Liberty (2004 )	3	3	9
Smith (2013 )	NE	5	NE
Wright et al. (2005 )	NE	NE	NE
Catanese et al. (1995 )	NE	5	NE
Khoshvaght et al. (2017 )	NE	NE	NE
Law et al. (2004 )	NE	NE	NE
**Studies with no effect from EC**			
Estudo 2 (Bochner et al. 1999)	6	4	24
Myrhaug et al. (2017 )	4	5	20
Myrhaug et al. (2019 )	4	5	20
Ödman and Öberg (2005 )	4	5	20
Ödman and Öberg (2006 )	4	5	20
Estudo 1 (Bochner et al. 1999)	4,5	4	18
Fase B (Blank et al. 2008)	1	2	2
Sigafoos et al. (2020) (Sigafoos et al. 1994)	NE	NE	NE
Hur (1997 )	NE	NE	NE

*Note:* This table has a total of 20 studies because the article by Bochner et al. (1999) deals with two studies with different practice times; the same occurred in the study by Blank et al. (2008), whose phases A and B have different practice times.

The quality analysis of the studies showed that seven (38.9%) were classified as having a low risk of bias, five (27.8%) with a moderate risk and six (33.3%) with a high risk of bias (Table 3). The item with the highest presence of bias risk was the pre‐intervention domain, particularly related to confounding bias. In 27.7% of the studies included in this review, confounding variables were either not adequately controlled or reported.

**TABLE 3:** Study quality classification by ROBINS‐I (11) and results of intervention with CE and the results of the intervention with EC.

	1	2	3	4	5	6	7	Classification	EC intervention results
**Low risk of bias**									
Sigafoos et al. (1994)	L	L	L	M	L	L	L	L	—
Smith (2013)	L	L	L	L	L	L	L	L	↑
Wright et al. (2005)	L	L	L	L	L	L	L	L	↑
Effgen and Chan (2010)	M	L	L	L	L	M	L	L	↑
Catanese et al. (1995)	M	M	L	L	L	L	L	L	↑
Ödman and Öberg (2005)	M	L	L	L	L	L	L	L	—
Myrhaug et al. (2014)	M	L	L	L	L	L	M	L	—
**Moderate risk of bias**									
Khoshvaght et al. (2017)	M	M	M	L	L	L	M	M	↑
Myrhaug et al. (2017)	M	M	L	L	L	L	M	M	—
Dalvand et al. (2009)	M	M	L	L	L	M	L	M	↑
Blank et al. (2008)	M	M	M	M	M	M	M	M	↑
Ilundáin and Fernández (2007)	M	M	L	L	L	L	M	M	↑
**High risk of bias**									
Bochner et al. (1999)	S	L	S	L	L	S	L	S	—
Hur (1997)	M	M	M	L	L	S	L	S	—
Coleman et al. (1995)	S	M	L	L	S	L	L	S	↑
Liberty (2004)	S	M	M	M	L	L	M	S	↑
Ödman and Öberg (2006)	S	L	L	L	L	L	M	S	—
Law et al. (2004)	S	L	L	L	M	M	M	S	↑

*Note:* Items: (1) Confusion bias; (2) Bias in the selection of participants for the study; (3) Bias in the classification of interventions; (4) Bias due to deviations from the intended interventions; (5) Bias due to lack of data; (6) Bias in the measurement of results; (7) Bias in the selection of the reported outcome. L (low risk of bias); M (moderate risk of bias); S (high risk of bias). Results: ‐, no effect for EC; ↑, positive effect from EC.

The motor assessment was conducted using 14 different instruments. Of the 18 articles included in this study, eight used more than one assessment tool (Table 4). Regarding the professional responsible for applying the tools, four studies reported that physiotherapists conducted the assessments, one study noted that neurologists and educators performed the assessments and two studies had occupational therapists as the evaluators. However, in 11 studies, the professional who conducted the assessment was not specified. The most used instrument was the Gross Motor Function Measure (GMFM), employed in eight studies. This was followed by the Paediatric Disability Assessment Inventory, used in four studies.

**TABLE 4:** Absolute and relative frequency of the motor assessment instrument used and of the subjects according to the types and topography of cerebral palsy in the results of the intervention with CE.

	Positive effects from EC		No effect from EC	
	Studies			
**Motor assessment instrument**	*n*	%	*n*	%
Battery of objective quantitative measurements of finger‐hand functions	1	6.3	0	0.0
Questionnaire for the measurement of activities of daily living	1	6.3	0	0.0
Vulpe Assessment Battery	2	12.5	0	0.0
Customer Development Assessment Report	1	6.3	0	0.0
Gross Motor Function Measure	4	25.0	4	30.8
The Uniform Performance Evaluation System	1	6.3	0	0.0
Lincoln–Oseretsky Scale	1	6.3	0	0.0
Scale of Achievement of Goals	2	12.5	0	0.0
Peabody Developmental Motor Scale	2	12.5	0	0.0
Timed Up and Go	1	6.3	0	0.0
Paediatric Disability Assessment Inventory	0	0.0	4	30.8
Paediatric Quality of Life Inventory	0	0.0	3	23.1
Functional Skills Assessment Scales	0	0.0	1	7.7
Adaptive Behaviour Scales of Vineland	0	0.0	1	7.7
	Subjects			
**Type and topography**	*n*	%	*n*	%
Spastic‐diplegia	109	60.9	32	40.0
Spastic‐hemiplegia	25	14.0	12	15.0
Spastic tetraparesis	16	8.9	0	0.0
Spastic‐tetraplegia	15	8.4	10	12.5
Dystonia	6	3.4	0	0.0
Ataxic	4	2.2	4	5.0
Athetoid	3	1.7	22	27.5
Spastic‐mixed	1	0.6	0	0.0

*Note:* Some studies used more than one motor assessment instrument; not all articles provided information on the type and topography of the cerebral palsy patient's injury.

Regarding the types and topography of CP, most studies investigated samples whose subjects were classified as spastic CP and diplegic and hemiplegic types (Table 4). This occurred both in studies that found positive effects of CE and in studies that did not find the effect of CE. It was also observed that among the studies that did not find the effects of CE, 32.5% of the sample consisted of subjects with athetoid or ataxic CP, and these types of CP appeared in only 3.9% of the samples of the studies where there was an effect of CE.

## 4 Discussion

This systematic and integrative literature review investigated interventions using CE for children with CP. Our analysis revealed a growing trend in CE intervention studies from 1995 to 2024, with Australia emerging as a prominent research hub. Most studies included relatively small sample sizes (21–30 participants), potentially reflecting recruitment challenges at individual study sites. Our findings suggest that CE interventions generally produce positive effects on motor performance in children and adolescents with CP. However, these results must be interpreted cautiously, considering several risks of bias factors, including practice duration, assessment tool selection and the specific type and topographical presentation of CP.

### 4.1 Effect of CE Intervention and the Methodological Quality of the Studies

The quality assessment revealed that most studies (*n* = 12; 66.5%) presented low or moderate risk of bias. Of these, eight studies (66.7%) (Catanese et al. 1995; Smith 2013; Wright et al. 2005; Effgen and Chan 2010; Khoshvaght et al. 2017; Dalvand et al. 2009; Blank et al. 2008; Ilundáin and Fernández 2007) demonstrated positive effects of CE on motor performance in children and adolescents with CP. Among studies with high risk of bias (*n* = 6; 34%), three (50%) (Coleman et al. 1995; Liberty 2004; Law et al. 2004) identified positive effects of CE intervention on motor improvement. Collectively, these results provide convincing support for the positive effect of CE intervention on motor performance in young people with CP.

Among studies with high risk of bias, several common methodological issues were identified. Coleman et al.'s (1995) study showed confounding bias due to lack of randomization in intervention and control group formation, and inadequate control of confounding factors (such as socioeconomic status and CP severity) in both implementation and data analysis. Similar risks were observed in Liberty's (2004) study, which failed to use randomization in group selection and did not control for confounding variables, including intervention intensity and children's individual characteristics. Law et al.'s (2004) study lacked participant randomization and applied uniform intervention across all participants; furthermore, the absence of outcome assessor blinding may have introduced measurement bias. These findings suggest that future intervention studies should properly control confounding variables (including intervention intensity, contextual and individual participant characteristics), implement random group assignment and ensure outcome assessor blinding. These methodological improvements would minimize bias risks and strengthen evidence regarding CE effectiveness in children with CP.

### 4.2 The Impact of the Practice Duration

Our findings suggest that practice duration plays a crucial role in CE interventions: Studies reporting higher weekly practice hours (mean = 25.17 h/week) demonstrated more positive CE intervention outcomes compared to those with lower weekly hours (mean = 17.71 h/week). Indeed, motor learning literature indicates that motor performance improvement is directly associated with practice quantity: Greater exposure to motor skill experiences leads to enhanced learning (Schmidt 1993). Based on our review, CE intervention with an average duration of 25 h/week appears to be a favourable practice amount for promoting motor performance improvements in children with CP.

It should be considered that meeting this average practice time may not be feasible in all contexts, as the time dedicated to the care of children and adolescents with CP can be affected by individual and contextual factors, such as age, degree of impairment and family socioeconomic status (Schmidt 1993). However, CE interventions with shorter weekly durations also showed positive effects (Dalvand et al. 2009; Coleman et al. 1995; Liberty 2004), and many studies did not report their intervention duration. This suggests caution in determining practice duration, as context, participants' clinical condition and intervention feasibility must be considered.

### 4.3 The Type of Assessment in Intervention Studies With CE

The assessment of motor skills in children with CP is central both in the planning of therapeutic and educational interventions, as well as in the context of research. A greater predominance of the use of the GMFM instrument was identified, used in eight studies (Myrhaug et al. 2019; Ödman and Öberg 2005; Ödman and Öberg 2006; Wright et al. 2005; Effgen and Chan 2010; Ilundáin and Fernández 2007; Law et al. 2004; Myrhaug et al. 2017); then the most used were the Paediatric Evaluation of Disability Inventory (PEDI), applied in five studies (Myrhaug et al. 2019; Ödman and Öberg 2005; Ödman and Öberg 2006; Wright et al. 2005; Myrhaug et al. 2017), the Vulpe Assessment Battery (VAB) applied in two studies (Catanese et al. 1995; Coleman et al. 1995) and the Goal Attainment Scaling (GAS) applied in two studies (Wright et al. 2005; Law et al. 2004).

The GMFM (Harvey 2017) is a validated instrument for assessing gross motor function in children aged 5 months to 16 years with CP and other motor conditions (Alotaibi et al. 2014). It measures abilities such as lying, rolling, sitting, crawling, kneeling, standing and walking, enabling monitoring of changes over time in rehabilitation settings; two versions exist: GMFM‐88, which provides comprehensive assessment through 88 items, and GMFM‐66, which employs Rasch analysis for more precise continuous measurements. However, both versions have limitations: GMFM‐8's length can be impractical in high‐demand settings, while GMFM‐66 requires specialized software, potentially limiting its use in resource‐constrained environments (Harvey 2017). Despite these limitations, GMFM remains a standard reference in motor assessment, though its selection should consider clinical context, available resources and intervention objectives.

The PEDI is a validated instrument (Berg et al. 2004) designed to assess functionality in children aged 6 months to 7 years, including those with CP and other congenital conditions. It comprises two sections—the Functional Skills Scale and the Caregiver Assistance Scale—evaluating children's competencies in three domains (self‐care, mobility and social function) and the required support level for daily tasks. The instrument can be implemented in clinical and educational settings, offering a contextual analysis of the child's daily activities rather than merely describing deficits or developmental milestones. Notably, PEDI enables parents and caregivers to better understand their children's capabilities (Kramer et al. 2016; Reid et al. 1994; Haley et al. 2010).

Despite its versatility, PEDI presents several limitations. The administration time can be prohibitive in clinical settings. Its application to children with severe CP may be limited as they might be unable to perform many assessed daily activities. Additionally, the scoring system, based on task completion, may inadequately reflect the difficulty level children face in specific tasks, such as arm movement or motor action coordination (Reid et al. 1994; Palisano et al. 1997).

Furthermore, PEDI assessments risk bias as they rely on parent/caregiver information, potentially compromising accuracy due to emotional factors or performance expectations that may overestimate children's abilities (Reid et al. 1994; Palisano et al. 1997). In response to the growing demand for more precise functional assessments, the Paediatric Evaluation of Disability Inventory–Computer Adaptive Test (PEDI‐CAT) (Haley et al. 2010) was developed, providing an adaptive, efficient and detailed evaluation of functional abilities in individuals aged 0–20 years, offering precise analysis of limitations and support needs and facilitating progress monitoring across clinical, educational and social contexts (Haley et al. 2010; Mancini et al. 2016).

The VAB (Vulpe 1977) is a validated instrument for assessing children aged 0–6 years, particularly those with specific developmental needs or delays, across clinical, educational or therapeutic settings. It evaluates cognitive, emotional and motor skills, including gross and fine motor abilities such as walking, running, drawing and object manipulation. Despite its widespread use, the VAB presents several limitations. It was not originally designed for children with CP, though adaptation is possible (Jain et al. 1994). The instrument may not comprehensively cover all motor development dimensions, potentially providing a limited view of a child's motor profile. Additional challenges include lengthy administration time, which may cause fatigue and affect results (Darrah et al. 2004), and the requirement for specialized training, particularly problematic in resource‐limited settings (Jain et al. 1994).

The Goal Attainment Scale (GAS) is an assessment tool designed to measure individual progress towards specific goals across all age groups. It is primarily used in therapeutic and educational interventions, distinguished by its ability to monitor progress through an individualized approach in paediatric contexts. GAS plays a crucial role in formulating motor, cognitive and social development goals, enabling healthcare professionals and educators to monitor children's progress and adjust interventions accordingly. Although not specifically designed for children with CP, its flexibility and goal customization capabilities make it applicable for monitoring progress in treatments for this population (Urach et al. 2019; Vu and Law 2012; Turner‐Stokes 2009). However, there are some limitations: the GAS observational format and individualized goal setting may introduce interpretation biases from both parents/caregivers and evaluators, potentially compromising standardization and result validity; also, GAS effectiveness depends heavily on active participant and evaluator engagement, which can be problematic in contexts with high turnover or low programme adherence (Steenbeek et al. 2007). Despite these limitations, GAS remains valuable for interventions prioritizing individualization and specific outcome measurements. To address its challenges, appropriate professional training and complementary assessment strategies are essential. In research, GAS can be particularly useful for studying personalized intervention efficacy in specific populations, provided its limitations are acknowledged and addressed (Vu and Law 2012).

### 4.4 Intervention Studies of CE, Observing the Typology and Topography of CP

Our results indicated that CE positively impacted motor skill improvement in children and adolescents with various types and topographies of CP. Although studies demonstrated considerable variety in types and topographies, spastic CP, in its diverse manifestations, was the most frequently observed type in studies included in this review. This type is among the small common and is characterized by increased muscle tone and rigidity, resulting from damage to brain areas controlling movement, thus affecting mobility, self‐care abilities and motor skill development. Regarding topography, diplegia was the most investigated. In this context, muscle weakness and spasticity predominantly affect the lower limbs, characterized by leg spasticity, walking difficulties, delayed motor skill development and reduced leg muscle strength (Wright et al. 2005).

Dalvand et al. (2009) effectively illustrate CE's impacts on this type and topography of CP. The authors evaluated different intervention methods' effects on improving daily activities in 45 children with spastic diplegic CP. Their results indicated that CE intervention was most effective, leading to improved walking balance and faster walking patterns, achieving 7 of 31 goals (23%) within 3 weeks—a notable achievement considering that most children with CP typically have goals set in 1‐year increments.

Although no included study specifically compared motor gains according to type and topography (data were used only for informational purposes), some authors report how these factors can impact their results. Coleman et al. (1995) emphasized that disability type and severity, presence or absence of associated impairments and family emotional well‐being can influence outcomes. Liberty's (2004) study results demonstrated that children with spastic quadriplegia and severe developmental delay in the experimental group showed significant functional skill gains compared to children with similar disabilities participating in the community program (control group). These findings collectively suggest that while CE demonstrates positive outcomes across various CP types and topographies, the effectiveness of interventions may vary depending on individual characteristics, highlighting the importance of considering both CP classification and associated factors when designing and implementing CE programmes.

Based on our findings, we propose several key implications for clinical practice and future research directions. For clinical practice, our results suggest that CE programmes should aim for approximately 25 h of weekly practice to optimize outcomes, while considering individual patient characteristics and available resources. The effectiveness of CE appears particularly promising for children with spastic CP, especially those with diplegic presentation, suggesting the need for tailored approaches based on CP type and topography. Future research should prioritize standardized protocols with clearly defined practice parameters, longer follow‐up periods and consistent outcome measures, particularly focusing on the GMFM as a primary assessment tool. We recommend that researchers document detailed intervention protocols, including practice intensity, frequency and duration, to enable better comparison across studies.

### 4.5 Strengths and Limitations of the Study

This systematic review presents some strengths that contribute to its scientific rigour and practical relevance. The comprehensive search strategy, encompassing multiple databases and a significant time span, allowed for a thorough examination of CE interventions in children with CP. The implementation of the ROBINS‐I tools for methodological quality assessment provided a systematic evaluation of bias risk across studies, strengthening the reliability of our findings. The detailed analysis of practice duration, assessment tools and CP types/topographies in relation to CE effectiveness offers valuable insights for both researchers and practitioners. Furthermore, the international scope of included studies provides a broad perspective on CE implementation across different cultural and healthcare contexts.

However, several limitations should be considered when interpreting our findings. Most included studies had relatively small sample sizes, which potentially limits statistical power and generalizability. The high heterogeneity in intervention protocols and assessment methods complicated direct comparisons between studies. Several studies lacked detailed information about practice duration, making it challenging to fully evaluate this crucial aspect. The review may be subject to publication bias as only published studies were included, and language restrictions (English, Portuguese and Spanish) may have excluded relevant studies in other languages. Additionally, the lack of long‐term follow‐up data in most studies limits our understanding of CE's sustained effects. The variable quality of included studies, with one‐third showing high risk of bias, necessitates cautious interpretation of some findings. These limitations highlight the need for larger, more standardized studies with longer follow‐up periods to strengthen the evidence base for CE interventions.

Although meta‐analysis is often valuable in systematic reviews, we opted for a qualitative synthesis approach due to several reasons. Our methodologically sound decision to employ qualitative synthesis rather than meta‐analysis addressed the inherent limitations of small sample sizes and insufficient statistical power across most studies (61.2% with ≤ 30 participants). But beyond that, there were other considerations as the substantial heterogeneity across included studies made statistical pooling problematic and potentially misleading. This heterogeneity was evident in multiple aspects: Intervention protocols varied considerably (2–40 weekly practice hours), assessment methods differed significantly (14 distinct instruments were used) and sample characteristics showed marked variation in CP types and topography. Furthermore, many studies lacked standardized reporting of intervention duration and complete quantitative data necessary for effect size calculations.

Given these limitations, we determined that a qualitative synthesis would better serve our primary aim of examining methodological aspects, practice time effects, assessment tools and CP characteristics that might influence intervention outcomes. This approach enabled a more nuanced understanding of these variables while maintaining scientific consistency in our analysis, providing meaningful insights that can guide more robust future investigations of CE effectiveness.

## 5 Conclusions

This systematic review provides substantial evidence supporting the effectiveness of CE as an intervention method for improving motor performance in children with CP. Key findings indicate that CE's effectiveness is significantly influenced by three main factors: practice duration, assessment methodology and CP type/topography. Studies implementing longer weekly practice hours (approximately 25 h/week) demonstrated more positive outcomes compared to those with shorter durations. The review also highlights the importance of appropriate assessment tool selection, with the GMFM and PEDI emerging as the most used instruments despite their limitations.

The analysis revealed that CE appears particularly effective for children with spastic CP, especially those with diplegic presentation, though positive outcomes were observed across various CP types and topographies. The methodological quality assessment showed that two‐thirds of the studies had low or moderate risk of bias, with those studies generally reporting positive CE effects, strengthening the evidence base for CE intervention.

These findings suggest that CE can be an effective intervention approach when properly implemented, though its success depends on adequate practice duration, appropriate assessment methods and consideration of individual patient characteristics. Future research should focus on standardizing intervention protocols, implementing longer follow‐up periods and investigating the specific factors that contribute to optimal outcomes in different CP populations.

## Author Contributions

N.N.S., L.M.D., M.P.S. and M.T.C. conceptualized and designed the study, designed the data collection, coordinated and supervised data collection, added important theoretical and methodological material and critically reviewed the manuscript. N.N.S., L.M.D. and M.T.C. collected data, made substantial contributions to research design, carried out the initial analyses, drafted the initial manuscript and critically reviewed the manuscript. N.N.S., A.L.O.T., A.A.A.S., D.C.A.O., A.B.D.S., A.H.P.F. and M.T.C. carried out the data analyses, added important theoretical and methodological material and critically reviewed the manuscript for important intellectual content. All authors approved the final manuscript as submitted and agree to be accountable for all aspects of the work.

## Ethics Statement

The authors have nothing to report.

## Conflicts of Interest

The authors declare no conflicts of interest.

## Data Availability

No primary data were generated for this integrative literature review. All data extracted from included studies and the synthesized analyses are presented within the article. Data extraction forms and additional methodological details are available from the corresponding author upon reasonable request. The review protocol was registered at PROSPERO (CRD42024578760).
